# Inequalities for *α*-fractional differentiable functions

**DOI:** 10.1186/s13660-017-1371-6

**Published:** 2017-04-28

**Authors:** Yu-Ming Chu, Muhammad Adil Khan, Tahir Ali, Sever Silvestru Dragomir

**Affiliations:** 1 Department of Mathematics, Huzhou University, Huzhou, 313000 China; 2 Department of Mathematics, University of Peshawar, Peshawar, 25000 Pakistan; 3 College of Engineering and Science, Victoria University, Melbourne, VIC 8001 Australia

**Keywords:** 26D15, 26A51, 26A33, convex function, Hermite-Hadamard inequality, fractional derivative, fractional integral, special mean, trapezoidal formula

## Abstract

In this article, we present an identity and several Hermite-Hadamard type inequalities for conformable fractional integrals. As applications, we establish some inequalities for certain special means of two positive real numbers and give the error estimations for the trapezoidal formula.

## Introduction

A real-valued function $\psi: \mathrm{I}\subseteq \mathbb{R}\rightarrow\mathbb{R}$ is said to be convex on I if the inequality

1.1 $$ \psi\bigl(\theta\xi+(1-\theta)\zeta\bigr)\leq\theta \psi(\xi)+(1-\theta)\psi( \zeta) $$

holds for all $\xi, \zeta\in\mathrm{I}$ and $\theta\in[0, 1]$. *ψ* is said to be concave on I if inequality (1.1) is reversed.

Let $\psi: \mathrm{I}\subseteq\mathbb{R} \rightarrow\mathbb{R}$ be a convex function on the interval I, and $c_{1}, c_{2} \in\mathrm{I}$ with $c_{1}< c_{2}$. Then the double inequality

1.2 $$ \psi \biggl(\frac{c_{1}+c_{2}}{2} \biggr)\leq\frac{1}{c_{2}-c_{1}} \int _{c_{1}}^{c_{2}}\psi(\xi)\,d\xi\leq\frac{\psi(c_{1})+\psi(c_{2})}{2} $$

is known in the literature as the Hermite-Hadamard inequality for convex functions [1–3]. Both inequalities hold in the reversed direction if *ψ* is concave on the interval I. In particular, many classical inequalities for means can be derived from (1.2) for appropriate particular selections of the function *ψ*.

Recently, the improvements, generalizations, refinements and applications for the Hermite-Hadamard inequality have attracted the attention of many researchers [4–22].

Dragomir and Agarwal [23] proved the following results connected with the right hand part of (1.2).

### Theorem 1.1

See [23], Lemma 2.1

*Let* $\psi:\mathrm{I}^{\circ}\subseteq\mathbb{R}\rightarrow \mathbb{R}$ *be a differentiable mapping on* $\mathrm{I}^{\circ}$. *Then the identity*

$$ \frac{\psi(c_{1})+\psi(c_{2})}{2}-\frac{1}{c_{2}-c_{1}} \int _{c_{1}}^{c_{2}}\psi(\xi)\,d\xi =\frac{c_{2}-c_{1}}{2} \int_{0}^{1}(1-2\theta)\psi^{\prime}\bigl( \theta c_{1}+(1-\theta)c_{2}\bigr)\,d\theta $$

*holds for all* $c_{1}, c_{2}\in\mathrm{I}^{\circ}$ *with* $c_{1}< c_{2}$ *if* $\psi^{\prime}\in\mathrm{L}[c_{1}, c_{2}]$, *where* $\mathrm{I}^{\circ}$ *denotes the interior of* I.

### Theorem 1.2

See [23], Theorem 2.2

*Let* $\psi:\mathrm{I}^{\circ}\subseteq\mathbb{R}\rightarrow \mathbb{R}$ *be a differentiable mapping on* $\mathrm{I}^{\circ}$. *Then the inequality*

$$ \biggl\vert \frac{\psi(c_{1})+\psi(c_{2})}{2}-\frac{1}{c_{2}-c_{1}} \int _{c_{1}}^{c_{2}} \psi(\xi)\,d\xi \biggr\vert \leq \frac{(c_{2}-c_{1})( \vert \psi^{\prime }(c_{1}) \vert + \vert \psi^{\prime}(c_{2}) \vert )}{8} $$

*holds for* $c_{1}, c_{2}\in\mathrm{I}^{\circ}$ *with* $c_{1}< c_{2}$ *if* $\vert \psi^{\prime} \vert $ *is convex on* $[c_{1}, c_{2}]$.

Making use of Theorem 1.1, Pearce and Pečarić [24] established Theorem 1.3 as follows.

### Theorem 1.3

See [24], Theorem 1

*Let* $c_{1}, c_{2}\in\mathrm{I}\subseteq\mathbb{R}$ *with* $c_{1}< c_{2}$, $\psi:\mathrm{I}^{\circ}\subseteq \mathbb{R}\rightarrow\mathbb{R}$ *be a differentiable mapping on* $\mathrm{I}^{\circ}$ *and* $q\geq1$. *Then the inequality*

$$ \biggl\vert \frac{\psi(c_{1})+\psi(c_{2})}{2}-\frac{1}{c_{2}-c_{1}} \int_{c_{1}}^{c_{2}}\psi(\xi)\,d\xi \biggr\vert \leq \frac{c_{2}-c_{1}}{4} \biggl[\frac{ \vert \psi^{\prime}(c_{1}) \vert ^{q}+ \vert \psi^{\prime}(c_{2}) \vert ^{q}}{2} \biggr]^{1/q} $$

*is valid if the mapping* $\vert \psi^{\prime} \vert ^{q}$ *is convex on the interval* $[c_{1}, c_{2}]$.

Next, we recall several elementary definitions and important results in the theory of conformable fractional calculus, which will be used throughout the article, we refer the interested reader to [25–32].

The conformable fractional derivative of order $0<\alpha\leq1$ for a function $\psi: (0, \infty)\rightarrow\mathbb{R}$ at $\xi>0$ is defined by

$$ \mathrm{D}_{\alpha}(\psi) (\xi)=\lim_{\epsilon\rightarrow 0} \frac{\psi(\xi+\epsilon\xi^{1-\alpha})-\psi(\xi)}{\epsilon}, $$

and the fractional derivative at 0 is defined as $\mathrm{D}_{\alpha}(\psi)(0)=\lim_{\xi\rightarrow 0^{+}}\mathrm{D}_{\alpha}(\psi)(\xi)$.

The (left) fractional derivative starting from $c_{1}$ of a function $\psi: [c_{1}, \infty)\rightarrow\mathbb{R}$ of order $0<\alpha \leq1$ is defined by

$$ \mathrm{D}_{\alpha}^{c_{1}}(\psi) (\xi)=\lim_{\epsilon\rightarrow 0} \frac{\psi(\xi+\epsilon (\xi-c_{1})^{1-\alpha})-\psi(\xi)}{\epsilon}, $$

and we write $\mathrm{D}_{\alpha}^{c_{1}}(\psi)=\mathrm{D}_{\alpha}^{0}(\psi)=\mathrm {D}_{\alpha}(\psi)$ if $c_{1}=0$. For more details see [26].

Let $\alpha\in(0, 1]$ and $\psi, \phi$ be *α*-differentiable at $\xi>0$. Then we have

1.3 $$\begin{aligned}& \frac{d_{\alpha}}{d_{\alpha}\xi} \bigl(\xi^{n} \bigr) =n\xi^{n-\alpha}, \quad n \in\mathbb{R}, \\& \frac{d_{\alpha}}{d_{\alpha}\xi}(c) =0, \quad c\in\mathbb{R}, \\& \frac{d_{\alpha}}{d_{\alpha}\xi} \bigl(c_{1} \psi(\xi)+ c_{2} \phi(\xi) \bigr) =c_{1} \frac{d_{\alpha}}{d_{\alpha}\xi}\bigl(\psi(\xi)\bigr)+c_{2} \frac{d_{\alpha}}{d_{\alpha}\xi}\bigl(\phi(\xi)\bigr), \quad c_{1}, c_{2} \in\mathbb{R}, \\& \frac{d_{\alpha}}{d_{\alpha}\xi} \bigl(\psi(\xi)\phi(\xi) \bigr) =\psi(\xi) \frac{d_{\alpha}}{d_{\alpha}\xi} \bigl(\phi(\xi)\bigr)+\phi(\xi) \frac{d_{\alpha}}{d_{\alpha}\xi}\bigl(\psi(\xi)\bigr), \\& \frac{d_{\alpha}}{d_{\alpha}\xi} \biggl(\frac{\psi(\xi)}{\phi(\xi)} \biggr) =\frac{\phi(\xi) \frac{d_{\alpha}}{d_{\alpha}\xi}(\psi(\xi))-\phi(\xi) \frac{d_{\alpha}}{d_{\alpha}\xi}(\psi(\xi))}{(\phi(\xi))^{2}}, \\& \frac{d_{\alpha}}{d_{\alpha}\xi} \bigl(\psi(\phi) (\xi) \bigr)=\psi'\bigl(\phi ( \xi)\bigr)\frac{d_{\alpha}}{d_{\alpha}\xi}\bigl(\phi(\xi)\bigr), \end{aligned}$$

where *ψ* is differentiable at $\phi(\xi)$ in equation (1.3). In particular,

$$ \frac{d_{\alpha}}{d_{\alpha}\xi} \bigl(\psi(\xi) \bigr)=\xi^{1-\alpha} \frac{d}{d\xi} \bigl(\psi(\xi)\bigr) $$

if *ψ* is differentiable.

Let $\alpha\in(0, 1] $ and $0\leq c_{1} < c_{2}$. A function $\psi : [c_{1}, c_{2}] \rightarrow\mathbb{R} $ is said to be *α*-fractional integrable on $[c_{1}, c_{2}]$ if the integral

$$ \int_{c_{1}}^{c_{2}}\psi(\xi)\,d_{\alpha}\xi= \int_{c_{1}}^{c_{2}}\psi(\xi )\xi^{\alpha-1}\,d\xi $$

exists and is finite. All the *α*-fractional integrable functions on $[c_{1}, c_{2}]$ are denoted by $\mathrm{L}_{\alpha}^{1}([c_{1}, c_{2}])$.

It is well known that

$$ \int_{c_{1}}^{c_{2}}\psi(\xi)\mathrm{D}_{\alpha}^{c_{1}}( \phi) (\xi )\,d_{\alpha}\xi=\psi\phi| _{c_{1}}^{c_{2}} - \int_{c_{1}}^{c_{2}}\phi(\xi)\mathrm{D}_{\alpha}^{c_{1}}( \psi) (\xi )\,d_{\alpha}\xi $$

if $\psi, \phi: [c_{1}, c_{2}] \rightarrow\mathbb{R}$ are two functions such that *ψϕ* is differentiable.

Very recently, Anderson [33] established a Hermite-Hadamard type inequality for fractional differentiable functions as follows.

### Theorem 1.4

*Let* $\alpha\in(0, 1]$ *and* $\psi: [c_{1}, c_{2}]\rightarrow\mathbb{R}$ *be an* *α*-*fractional differentiable function*. *Then the inequality*

1.4 $$ \frac{\alpha}{c_{2}^{\alpha}-c_{1}^{\alpha}} \int_{c_{1}}^{c_{2}}\psi(\xi )\,d_{\alpha}\xi\leq \frac{\psi(c_{1})+\psi(c_{2})}{2} $$

*holds if* $\mathrm{D}_{\alpha}(\psi)$ *is increasing on* $[c_{1}, c_{2}]$. *Moreover*, *if the function* *ψ* *is decreasing on* $[c_{1}, c_{2}]$, *then one has*

1.5 $$ \psi \biggl(\frac{c_{1}+c_{2}}{2} \biggr)\leq\frac{\alpha}{c_{2}^{\alpha }-c_{1}^{\alpha}} \int_{c_{1}}^{c_{2}}\psi(\xi)\,d_{\alpha}\xi. $$

### Remark 1.5

We clearly see that inequalities (1.4) and (1.5) reduce to inequality (1.2) if $\alpha=1$.

The main purpose of the article is to present an identity and several Hermite-Hadamard type inequalities for conformable fractional integrals, establish some inequalities for certain special means of two positive real numbers and give the error estimations for the trapezoidal formula.

## Main results

In order to prove our main results we need a lemma, which we present in this section.

### Lemma 2.1

*Let* $\alpha\in(0, 1]$, $c_{1}, c_{2} \in\mathbb{R}$ *with* $0\leq c_{1}< c_{2}$ *and* $\psi:[c_{1}, c_{2}]\rightarrow\mathbb {R}$ *be an* *α*-*fractional differentiable function on* $(c_{1}, c_{2})$. *Then the identity*

$$\begin{aligned} &\frac{\psi(c_{1})+\psi(c_{2})}{2}-\frac{\alpha}{c_{2}^{\alpha }-c_{1}^{\alpha}} \int_{c_{1}}^{c_{2}}\psi(\xi)\,d_{\alpha}\xi \\ &\quad =\frac{(c_{2}-c_{1})}{2(c_{2}^{\alpha}-c_{1}^{\alpha})} \biggl[ \int _{0}^{1} \bigl(\bigl(\theta c_{1}+(1- \theta)c_{2}\bigr)^{2\alpha-1}-c_{2}^{\alpha}\bigl( \theta c_{1}+(1-\theta)c_{2}\bigr)^{\alpha-1} \bigr) \\ &\quad\quad{} \times\mathrm{D}_{\alpha}(\psi) \bigl(\theta c_{1}+(1- \theta)c_{2}\bigr)\theta^{1-\alpha}\,d_{\alpha}\theta \\ & \quad\quad{} + \int_{0}^{1} \bigl(\bigl(\theta c_{1}+(1- \theta)c_{2}\bigr)^{2\alpha-1}-c_{1}^{\alpha}\bigl( \theta c_{1}+(1-\theta)c_{2}\bigr)^{\alpha-1} \bigr) \\ &\quad\quad{} \times\mathrm{D}_{\alpha}(\psi) \bigl(\theta c_{1}+(1- \theta)c_{2}\bigr)\theta^{1-\alpha}\,d_{\alpha}\theta \biggr] \end{aligned}$$

*holds if* $\mathrm{D}_{\alpha}(\psi)\in \mathrm{L}_{\alpha}^{1}([c_{1}, c_{2}])$.

### Proof

Let $\xi=\theta c_{1}+(1-\theta)c_{2}$. Then making use of integration by parts, we get

2.1 $$\begin{aligned} & \int_{0}^{1} \bigl(\bigl(\theta c_{1}+(1- \theta)c_{2}\bigr)^{2\alpha-1}-c_{2}^{\alpha}\bigl( \theta c_{1}+(1-\theta)c_{2}\bigr)^{\alpha-1} \bigr) \mathrm{D} _{\alpha}(\psi) \bigl(\theta c_{1}+(1- \theta)c_{2}\bigr)\,d\theta \\ &\quad\quad{} + \int_{0}^{1} \bigl(\bigl(\theta c_{1}+(1- \theta)c_{2}\bigr)^{2\alpha-1}-c_{1}^{\alpha}\bigl( \theta c_{1}+(1-\theta)c_{2}\bigr)^{\alpha-1} \bigr) \mathrm{D} _{\alpha}(\psi) \bigl(\theta c_{1}+(1- \theta)c_{2}\bigr)\,d\theta \\ &\quad = \int_{0}^{1} \bigl(\bigl(\theta c_{1}+(1- \theta)c_{2}\bigr)^{\alpha}-c_{2}^{\alpha} \bigr)\psi'\bigl(\theta c_{1}+(1-\theta)c_{2} \bigr)\,d\theta \\ &\quad\quad{} + \int_{0}^{1} \bigl(\bigl(\theta c_{1}+(1- \theta)c_{2}\bigr)^{\alpha}-c_{1}^{\alpha} \bigr) \psi'\bigl(\theta c_{1}+(1-\theta)c_{2}\bigr)\,d\theta \\ &\quad = \bigl(\bigl(\theta c_{1}+(1-\theta)c_{2} \bigr)^{\alpha}-c_{2}^{\alpha} \bigr)\frac{\psi(\theta c_{1}+(1-\theta)c_{2})}{c_{1}-c_{2}} \bigg| _{0}^{1} \\ &\quad\quad{} - \int_{0}^{1}\alpha\bigl(\theta c_{1}+(1- \theta)c_{2}\bigr)^{\alpha-1}(c_{1}-c_{2}) \frac{\psi(\theta c_{1}+(1-\theta)c_{2})}{c_{1}-c_{2}}\,d\theta \\ &\quad\quad{} + \bigl(\bigl(\theta c_{1}+(1-\theta)c_{2} \bigr)^{\alpha}-c_{1}^{\alpha} \bigr)\frac{\psi(\theta c_{1}+(1-\theta)c_{2})}{c_{1}-c_{2}} \bigg| _{0}^{1} \\ &\quad\quad{} - \int_{0}^{1}\alpha\bigl(\theta c_{1}+(1- \theta)c_{2}\bigr)^{\alpha-1}(c_{1}-c_{2}) \frac{\psi(\theta c_{1}+(1-\theta)c_{2})}{c_{1}-c_{2}}\,d\theta \\ &\quad =\frac{1}{c_{2}-c_{1}} \biggl[\bigl(c_{2}^{\alpha}-c_{1}^{\alpha} \bigr)\psi (c_{1})-\alpha \int_{c_{1}}^{c_{2}}\psi(\xi)\,d_{\alpha}\xi \biggr] \\ &\quad\quad{} +\frac{1}{c_{2}-c_{1}} \biggl[\bigl(c_{2}^{\alpha}-c_{1}^{\alpha} \bigr)\psi(c_{2}) -\alpha \int_{c_{1}}^{c_{2}}\psi(\xi)\,d_{\alpha}\xi \biggr] \\ &\quad =\frac{c_{2}^{\alpha}-c_{1}^{\alpha}}{c_{2}-c_{1}} \bigl(\psi(c_{1})+\psi(c_{2}) \bigr) - \frac{2\alpha}{c_{2}-c_{1}} \int_{c_{1}}^{c_{2}}\psi(\xi)\,d_{\alpha}\xi. \end{aligned}$$

Therefore, Lemma 2.1 follows easily from (2.1). □

### Remark 2.2

We clearly see that the identity given in Lemma 2.1 reduces to the identity given in Theorem 1.1 if $\alpha=1$.

### Theorem 2.3

*Let* $\alpha\in(0, 1]$, $c_{1}, c_{2}\in\mathbb{R}$ *with* $0\leq c_{1} < c_{2}$ *and* $\psi:[c_{1}, c_{2}] \rightarrow\mathbb{R}$ *be an* *α*-*differentiable function*. *Then the inequality*

2.2 $$\begin{aligned} & \biggl\vert \frac{\psi(c_{1})+\psi(c_{2})}{2}-\frac{\alpha }{c_{2}^{\alpha}-c_{1}^{\alpha}} \int_{c_{1}}^{c_{2}}\psi(\xi)\,d_{\alpha }\xi \biggr\vert \\ &\quad \leq\frac{c_{2}-c_{1}}{2(c_{2}^{\alpha}-c_{1}^{\alpha})} \biggl[\frac{( \vert \psi^{\prime}(c_{1}) \vert + \vert \psi ^{\prime}(c_{2}) \vert ) (5c_{2}^{\alpha}-7c_{1}^{\alpha}+c_{1}c_{2}^{\alpha -1}+c_{1}^{\alpha-1}c_{2} )}{12} \biggr] \end{aligned}$$

*holds if* $\mathrm{D} _{\alpha}(\psi)\in \mathrm{L}_{\alpha}^{1}([c_{1}, c_{2}])$ *and* $\vert \psi^{\prime } \vert $ *is convex on* $[c_{1}, c_{2}]$.

### Proof

It follows from Lemma 2.1 and the convexities of the functions $\xi\rightarrow\xi^{\alpha-1}$ and $\xi\rightarrow-\xi^{\alpha}$ on $(0, \infty)$ together with the convexity of $\vert \psi^{\prime } \vert $ on $[c_{1}, c_{2}]$ that

$$\begin{aligned}& \biggl\vert \frac{\psi(c_{1})+\psi(c_{2})}{2}-\frac{\alpha }{c_{2}^{\alpha}-c_{1}^{\alpha}} \int_{c_{1}}^{c_{2}}\psi(\xi)\,d_{\alpha }\xi \biggr\vert \\& \quad \leq\frac{c_{2}-c_{1}}{2(c_{2}^{\alpha}-c_{1}^{\alpha})} \biggl[ \int _{0}^{1} \bigl(\bigl(\theta c_{1}+(1- \theta)c_{2}\bigr)^{\alpha}-c_{1}^{\alpha} \bigr) \bigl\vert \psi ^{\prime}\bigl(\theta c_{1}+(1- \theta)c_{2}\bigr) \bigr\vert \,d\theta \\& \quad\quad{} + \int_{0}^{1} \bigl(c_{2}^{\alpha}- \bigl(\theta c_{1}+(1-\theta)c_{2}\bigr)^{\alpha} \bigr) \bigl\vert \psi^{\prime}\bigl(\theta c_{1}+(1- \theta)c_{2}\bigr) \bigr\vert \,d\theta \biggr] \\& \quad \leq\frac{c_{2}-c_{1}}{2(c_{2}^{\alpha}-c_{1}^{\alpha})} \biggl[ \int _{0}^{1} \bigl(\bigl(\theta c_{1}+(1- \theta)c_{2}\bigr)^{\alpha-1}\bigl(\theta c_{1}+(1- \theta)c_{2}\bigr)-c_{1}^{\alpha} \bigr) \bigl\vert \psi^{\prime }\bigl(\theta c_{1}+(1-\theta)c_{2}\bigr) \bigr\vert \,d\theta \\& \quad\quad{} + \int_{0}^{1} \bigl(c_{2}^{\alpha}- \bigl((1-\theta)c_{1}^{\alpha}+\theta c_{2}^{\alpha} \bigr) \bigr) \bigl\vert \psi^{\prime}\bigl(\theta c_{1}+(1- \theta)c_{2}\bigr) \bigr\vert \,d\theta \biggr] \\& \quad \leq\frac{c_{2}-c_{1}}{2(c_{2}^{\alpha}-c_{1}^{\alpha})} \biggl[ \int _{0}^{1} \bigl(\bigl((1-\theta)c_{1}^{\alpha-1}+ \theta c_{2}^{\alpha-1}\bigr) \bigl(\theta c_{1}+(1- \theta)c_{2}\bigr)-c_{1}^{\alpha} \bigr) \bigl\vert \psi^{\prime }\bigl(\theta c_{1}+(1-\theta)c_{2}\bigr) \bigr\vert \,d\theta \\& \quad\quad{} + \int_{0}^{1} \bigl(c_{2}^{\alpha}- \bigl((1-\theta)c_{1}^{\alpha}+\theta c_{2}^{\alpha} \bigr) \bigr) \bigl\vert \psi^{\prime}\bigl(\theta c_{1}+(1- \theta)c_{2}\bigr) \bigr\vert \,d\theta \biggr] \\& \quad \leq\frac{c_{2}-c_{1}}{2(c_{2}^{\alpha}-c_{1}^{\alpha})} \biggl[ \int _{0}^{1} \bigl(\bigl((1-\theta)c_{1}^{\alpha-1}+ \theta c_{2}^{\alpha-1}\bigr) \bigl(\theta c_{1}+(1- \theta)c_{2}\bigr)-c_{1}^{\alpha} \bigr) \\& \quad\quad{}\times \bigl[(1- \theta) \bigl\vert \psi^{\prime}(c_{1}) \bigr\vert +\theta \bigl\vert \psi'(c_{2}) \bigr\vert \bigr]\,d\theta \\& \quad\quad{} + \int_{0}^{1} \bigl(c_{2}^{\alpha}- \bigl((1-\theta)c_{1}^{\alpha}+\theta c_{2}^{\alpha} \bigr) \bigr) \bigl[(1-\theta) \bigl\vert \psi^{\prime }(c_{1}) \bigr\vert +\theta \bigl\vert \psi'(c_{2}) \bigr\vert \bigr]\,d\theta \biggr] \\& \quad =\frac{c_{2}-c_{1}}{c_{2}^{\alpha}-c_{1}^{\alpha}} \biggl[\frac {1}{4}c_{1}^{\alpha} \bigl\vert \psi^{\prime}(c_{1}) \bigr\vert + \frac{1}{12}c_{1}^{\alpha} \bigl\vert \psi^{\prime}(c_{2}) \bigr\vert +\frac{1}{12}c_{1}c_{2}^{\alpha-1} \bigl\vert \psi^{\prime}(c_{1}) \bigr\vert + \frac{1}{12}c_{1}c_{2}^{\alpha-1} \bigl\vert \psi^{\prime}(c_{2}) \bigr\vert \\& \quad\quad{} +\frac{1}{12}c_{1}^{\alpha-1}c_{2} \bigl\vert \psi^{\prime }(c_{1}) \bigr\vert +\frac{1}{12}c_{1}^{\alpha-1}c_{2} \bigl\vert \psi^{\prime}(c_{2}) \bigr\vert +\frac{1}{12}c_{2}^{\alpha} \bigl\vert \psi^{\prime}(c_{1}) \bigr\vert + \frac{1}{4}c_{2}^{\alpha} \bigl\vert \psi^{\prime}(c_{2}) \bigr\vert \\& \quad\quad{} -\frac{1}{2}c_{1}^{\alpha} \bigl\vert \psi^{\prime}(c_{1}) \bigr\vert - \frac{1}{2}c_{1}^{\alpha} \bigl\vert \psi^{\prime}(c_{2}) \bigr\vert +\frac{1}{2}c_{2}^{\alpha} \bigl\vert \psi^{\prime}(c_{1}) \bigr\vert +\frac{1}{2}c_{2}^{\alpha} \bigl\vert \psi^{\prime}(c_{2}) \bigr\vert - \frac{1}{3}c_{1}^{\alpha} \bigl\vert \psi^{\prime}(c_{1}) \bigr\vert \\& \quad\quad{} -\frac{1}{6}c_{1}^{\alpha} \bigl\vert \psi^{\prime}(c_{2}) \bigr\vert - \frac{1}{6}c_{2}^{\alpha} \bigl\vert \psi^{\prime}(c_{1}) \bigr\vert -\frac{1}{3}c_{2}^{\alpha} \bigl\vert \psi^{\prime}(c_{2}) \bigr\vert \biggr] \\& \quad =\frac{c_{2}-c_{1}}{2(c_{2}^{\alpha}-c_{1}^{\alpha})} \biggl[\frac{( \vert \psi^{\prime}(c_{1}) \vert + \vert \psi ^{\prime}(c_{2}) \vert ) (5c_{2}^{\alpha}-7c_{1}^{\alpha}+c_{1}c_{2}^{\alpha -1}+c_{1}^{\alpha-1}c_{2} )}{12} \biggr]. \end{aligned}$$

 □

### Remark 2.4

Let $\alpha=1$. Then inequality (2.2) becomes

$$ \biggl\vert \frac{\psi(c_{1})+\psi(c_{2})}{2}-\frac{1}{c_{2}-c_{1}} \int _{c_{1}}^{c_{2}}\psi(\xi)\,d\xi \biggr\vert \leq \frac{c_{2}-c_{1}}{4} \bigl[ \bigl\vert \psi^{\prime}(c_{1}) \bigr\vert + \bigl\vert \psi^{\prime}(c_{2}) \bigr\vert \bigr]. $$

### Theorem 2.5

*Let* $\alpha\in(0, 1]$, $q>1$, $c_{1}, c_{2}\in\mathbb{R}$ *with* $0\leq c_{1} < c_{2}$ *and* $\psi:[c_{1}, c_{2}] \rightarrow \mathbb{R}$ *be an* *α*-*differentiable function on* $(c_{1}, c_{2})$. *Then the inequality*

2.3 $$\begin{aligned} & \biggl\vert \frac{\psi(c_{1})+\psi(c_{2})}{2}-\frac{\alpha }{c_{2}^{\alpha}-c_{1}^{\alpha}} \int_{c_{1}}^{c_{2}}\psi(\xi)\,d_{\alpha }\xi \biggr\vert \\ &\quad \leq\frac{c_{2}-c_{1}}{2(c_{2}^{\alpha}-c_{1}^{\alpha})} \bigl[ \bigl(\mathrm{A}_{1}(\alpha) \bigr)^{1-1/q} \bigl\{ \mathrm {A}_{2}(\alpha) \bigl\vert \psi^{\prime}(c_{1}) \bigr\vert ^{q}+ \mathrm{A}_{3} (\alpha) \bigl\vert \psi^{\prime}(c_{2}) \bigr\vert ^{q} \bigr\} ^{1/q} \\ &\quad\quad{} + \bigl(\mathrm{B}_{1}(\alpha) \bigr)^{1-1/q} \bigl\{ \mathrm {B}_{2}(\alpha) \bigl\vert \psi^{\prime}(c_{1}) \bigr\vert ^{q} +\mathrm{B}_{3}(\alpha) \bigl\vert \psi^{\prime}(c_{2}) \bigr\vert ^{q} \bigr\} ^{1/q} \bigr] \end{aligned}$$

*is valid if* $\mathrm{D}_{\alpha}(\psi)\in \mathrm{L}_{\alpha}^{1}([c_{1}, c_{2}])$ *and* $\vert \psi^{\prime } \vert ^{q}$ *is convex on* $[c_{1}, c_{2}]$, *where*

$$\begin{aligned}& \mathrm{A}_{1}(\alpha)= \biggl[\frac{c_{1}^{\alpha+1}-c_{2}^{\alpha +1}}{(\alpha+1)(c_{1}-c_{2})} \biggr]-c_{1}^{\alpha},\quad \quad \mathrm{B}_{1}( \alpha)=c_{2}^{\alpha}- \biggl[\frac{c_{1}^{\alpha +1}-c_{2}^{\alpha+1}}{(\alpha+1)(c_{1}-c_{2})} \biggr], \\ & \mathrm{A}_{2}(\alpha)= \biggl[\frac{-c_{2}^{\alpha+1}}{(\alpha+1)(c_{1}-c_{2})}+ \frac{c_{1}^{\alpha+2}-c_{2}^{\alpha+2}}{(\alpha+1)(\alpha +2)(c_{1}-c_{2})^{2}}-\frac{c_{1}^{\alpha}}{2} \biggr], \\ & \mathrm{B}_{2}(\alpha)= \biggl[\frac{c_{2}^{\alpha}}{2}+\frac {c_{2}^{\alpha+1}}{(\alpha+1)(c_{1}-c_{2})} +\frac{c_{1}^{\alpha+2}-c_{2}^{\alpha+2}}{(\alpha+1)(\alpha +2)(c_{1}-c_{2})^{2}} \biggr], \\ & \mathrm{A}_{3}(\alpha)= \biggl[\frac{c_{1}^{\alpha+1}}{(\alpha+1)(c_{1}-c_{2})} - \frac{c_{1}^{\alpha+2}-c_{2}^{\alpha+2}}{(\alpha+1)(\alpha +2)(c_{1}-c_{2})^{2}}-\frac{c_{1}^{\alpha}}{2} \biggr], \\ & \mathrm{B}_{3}(\alpha)= \biggl[\frac{c_{2}^{\alpha}}{2}-\frac {c_{1}^{\alpha+1}}{(\alpha+1)(c_{1}-c_{2})}+ \frac{c_{1}^{\alpha+2}-c_{2}^{\alpha+2}}{(\alpha+1)(\alpha +2)(c_{1}-c_{2})^{2}} \biggr]. \end{aligned}$$

### Proof

From Lemma 2.1 and the well-known Hölder mean inequality together with the convexity of $\vert \psi^{\prime} \vert ^{q}$ on the interval $[c_{1}, c_{2}]$ we clearly see that

2.4 $$\begin{aligned} & \biggl\vert \frac{\psi(c_{1})+\psi(c_{2})}{2}-\frac{\alpha }{c_{2}^{\alpha}-c_{1}^{\alpha}} \int_{c_{1}}^{c_{2}}\psi(\xi)\,d_{\alpha }\xi \biggr\vert \\ &\quad = \biggl\vert \frac{c_{2}-c_{1}}{2(c_{2}^{\alpha}-c_{1}^{\alpha})} \biggl[ \int_{0}^{1} \bigl(\bigl(\theta c_{1}+(1- \theta)c_{2}\bigr)^{2\alpha-1}-c_{1}^{\alpha}\bigl( \theta c_{1}+(1-\theta)c_{2}\bigr)^{\alpha-1} \bigr) \\ &\quad\quad{}\times \mathrm{D}_{\alpha}(\psi) \bigl(\theta c_{1}+(1- \theta)c_{2}\bigr)\,d\theta \\ &\quad\quad{} + \int_{0}^{1} \bigl(\bigl(\theta c_{1}+(1- \theta)c_{2}\bigr)^{2\alpha-1}-c_{2}^{\alpha}\bigl( \theta c_{1}+(1-\theta)c_{2}\bigr)^{\alpha-1} \bigr) \mathrm{D}_{\alpha}(\psi) \bigl(\theta c_{1}+(1- \theta)c_{2}\bigr)\,d\theta \biggr] \biggr\vert \\ &\quad \leq\frac{c_{2}-c_{1}}{2(c_{2}^{\alpha}-c_{1}^{\alpha})} \biggl[ \int _{0}^{1} \bigl(\bigl(\theta c_{1}+(1- \theta)c_{2}\bigr)^{\alpha}-c_{1}^{\alpha} \bigr) \bigl\vert \psi ^{\prime}\bigl(\theta c_{1}+(1- \theta)c_{2}\bigr) \bigr\vert \,d\theta \\ &\quad\quad{} + \int_{0}^{1} \bigl(c_{2}^{\alpha}- \bigl(\theta c_{1}+(1-\theta)c_{2}\bigr)^{\alpha} \bigr) \bigl\vert \psi^{\prime}\bigl(\theta c_{1}+(1- \theta)c_{2}\bigr) \bigr\vert \,d\theta \biggr], \end{aligned}$$

2.5 $$\begin{aligned} & \int_{0}^{1} \bigl(\bigl(\theta c_{1}+(1- \theta)c_{2}\bigr)^{\alpha}-c_{1}^{\alpha} \bigr) \bigl\vert \psi ^{\prime}\bigl(\theta c_{1}+(1- \theta)c_{2}\bigr) \bigr\vert \,d\theta \\ &\quad \leq \biggl( \int_{0}^{1} \bigl(\bigl(\theta c_{1}+(1- \theta)c_{2}\bigr)^{\alpha}-c_{1}^{\alpha} \bigr)\,d\theta \biggr)^{1-1/q} \\ &\quad\quad{} \times \biggl( \int_{0}^{1} \bigl(\bigl(\theta c_{1}+(1- \theta)c_{2}\bigr)^{\alpha}-c_{1}^{\alpha} \bigr) \bigl\vert \psi '\bigl(\theta c_{1}+(1- \theta)c_{2}\bigr) \bigr\vert ^{q}\,d\theta \biggr)^{1/q}, \end{aligned}$$

2.6 $$\begin{aligned} & \int_{0}^{1} \bigl(c_{2}^{\alpha}- \bigl(\theta c_{1}+(1-\theta)c_{2}\bigr)^{\alpha} \bigr) \bigl\vert \psi^{\prime}\bigl(\theta c_{1}+(1- \theta)c_{2}\bigr) \bigr\vert \,d\theta \\ &\quad \leq \biggl( \int_{0}^{1} \bigl( c_{2}^{\alpha}- \bigl(\theta c_{1}+(1-\theta)c_{2}\bigr)^{\alpha} \bigr)\,d\theta \biggr)^{1-1/q} \\ &\quad\quad{} \times \biggl( \int_{0}^{1} \bigl(c_{2}^{\alpha}- \bigl(\theta c_{1}+(1-\theta)c_{2}\bigr)^{\alpha} \bigr) \bigl\vert \psi^{\prime}\bigl(\theta c_{1}+(1- \theta)c_{2}\bigr) \bigr\vert ^{q}\,d\theta \biggr)^{1/q}, \end{aligned}$$

2.7 $$\begin{aligned} & \int_{0}^{1} \bigl(\bigl(\theta c_{1}+(1- \theta)c_{2}\bigr)^{\alpha}-c_{1}^{\alpha} \bigr) \bigl\vert \psi ^{\prime}\bigl(\theta c_{1}+(1- \theta)c_{2}\bigr) \bigr\vert ^{q}\,d\theta \\ &\quad \leq \int_{0}^{1} \bigl(\bigl(\theta c_{1}+(1- \theta)c_{2}\bigr)^{\alpha}-c_{1}^{\alpha} \bigr) \bigl[(1-\theta) \bigl\vert \psi^{\prime}(c_{1}) \bigr\vert ^{q}+\theta \bigl\vert \psi^{\prime }(c_{2}) \bigr\vert ^{q} \bigr]\,d\theta \\ &\quad = \bigl\vert \psi^{\prime}(c_{1}) \bigr\vert ^{q} \int_{0}^{1} \bigl(\bigl(\theta c_{1}+(1- \theta)c_{2}\bigr)^{\alpha}-c_{1}^{\alpha} \bigr) (1-\theta)\,d\theta \\ &\quad\quad{} + \bigl\vert \psi^{\prime}(c_{2}) \bigr\vert ^{q} \int_{0}^{1} \bigl(\bigl(\theta c_{1}+(1- \theta)c_{2}\bigr)^{\alpha}-c_{1}^{\alpha} \bigr) \theta \,d\theta \\ &\quad = \bigl\vert \psi^{\prime}(c_{1}) \bigr\vert ^{q} \biggl[\frac {-c_{2}^{\alpha+1}}{(\alpha+1)(c_{1}-c_{2})}+ \frac{c_{1}^{\alpha+2}-c_{2}^{\alpha+2}}{(\alpha+1)(\alpha +2)(c_{1}-c_{2})^{2}}-\frac{c_{1}^{\alpha}}{2} \biggr] \\ &\quad\quad{} + \bigl\vert \psi^{\prime}(c_{2}) \bigr\vert ^{q} \biggl[\frac{\alpha +1}{(\alpha+1)(c_{1}-c_{2})} -\frac{c_{1}^{\alpha+2}-c_{2}^{\alpha+2}}{(\alpha+1)(\alpha +2)(c_{1}-c_{2})^{2}}-\frac{c_{1}^{\alpha}}{2} \biggr], \end{aligned}$$

2.8 $$\begin{aligned} & \int_{0}^{1} \bigl(c_{2}^{\alpha}- \bigl(\theta c_{1}+(1-\theta)c_{2}\bigr)^{\alpha} \bigr) \bigl\vert \psi^{\prime}\bigl(\theta c_{1}+(1- \theta)c_{2}\bigr) \bigr\vert ^{q}\,d\theta \\ &\quad \leq \int_{0}^{1} \bigl(c_{2}^{\alpha}- \bigl(\theta c_{1}+(1-\theta)c_{2}\bigr)^{\alpha} \bigr) \bigl[(1-\theta) \bigl\vert \psi ^{\prime}(c_{1}) \bigr\vert ^{q}+\theta \bigl\vert \psi^{\prime }(c_{2}) \bigr\vert ^{q} \bigr]\,d\theta \\ &\quad = \bigl\vert \psi^{\prime}(c_{1}) \bigr\vert ^{q} \int_{0}^{1} \bigl(c_{2}^{\alpha}- \bigl(\theta c_{1}+(1-\theta)c_{2}\bigr)^{\alpha} \bigr) (1-\theta)\,d\theta \\ &\quad\quad{} + \bigl\vert \psi^{\prime}(c_{2}) \bigr\vert ^{q} \int_{0}^{1} \bigl(c_{2}^{\alpha}- \bigl(\theta c_{1}+(1-\theta)c_{2}\bigr)^{\alpha} \bigr)\theta \,d\theta \\ &\quad = \bigl\vert \psi^{\prime}(c_{1}) \bigr\vert ^{q} \biggl[\frac {c_{2}^{\alpha}}{2}+\frac{c_{2}^{\alpha+1}}{(\alpha+1) (c_{1}-c_{2})}+\frac{c_{1}^{\alpha+2}-c_{2}^{\alpha+2}}{(\alpha +1)(\alpha+2)(c_{1}-c_{2})^{2}} \biggr] \\ &\quad\quad{} + \bigl\vert \psi^{\prime}(c_{2}) \bigr\vert ^{q} \biggl[\frac {c_{2}^{\alpha}}{2}-\frac{c_{1}^{\alpha+1}}{ (\alpha+1)(c_{1}-c_{2})}+\frac{c_{1}^{\alpha+2}-c_{2}^{\alpha +2}}{(\alpha+1)(\alpha+2)(c_{1}-c_{2})^{2}} \biggr]. \end{aligned}$$

Therefore, inequality (2.3) follows easily from (2.4)-(2.8). □

### Remark 2.6

Let $\alpha=1$. Then inequality (2.3) becomes

$$\begin{aligned} & \biggl\vert \frac{\psi(c_{1})+\psi(c_{2})}{2}-\frac{1}{c_{2}-c_{1}} \int _{c_{1}}^{c_{2}}\psi(\xi)\,d\xi \biggr\vert \\ &\quad \leq\frac{1}{2} \biggl(\frac{c_{2}-c_{1}}{2} \biggr)^{1-1/q} \bigl[ \bigl\{ \mathrm{A}_{2}(1) \bigl\vert \psi^{\prime}(c_{1}) \bigr\vert ^{q}+ \mathrm{A}_{3}(1) \bigl\vert \psi^{\prime}(c_{2}) \bigr\vert ^{q} \bigr\} ^{1/q} \\ &\quad\quad{} + \bigl\{ \mathrm{B}_{2}(1) \bigl\vert \psi^{\prime}(c_{1}) \bigr\vert ^{q}+\mathrm{B}_{3}(1) \bigl\vert \psi^{\prime}(c_{2}) \bigr\vert ^{q} \bigr\} ^{1/q} \bigr] \end{aligned}$$

with

$$\begin{aligned}& \mathrm{A}_{2}(1)=\frac{c_{2}-c_{1}}{3},\quad\quad \mathrm{B}_{2}(1)= \frac {(c_{1}+c_{2})^{2}+2c_{1}c_{2}}{6(c_{1}-c_{2})}, \\ & \mathrm{A}_{3}(1)=\frac{c_{2}-c_{1}}{6}, \quad \quad \mathrm{B}_{3}(1)= \frac{c_{2}-c_{1}}{3}. \end{aligned}$$

### Theorem 2.7

*Let* $\alpha\in(0, 1]$, $q>1$, $c_{1}, c_{2}\in\mathbb{R} $ *with* $0\leq c_{1} < c_{2}$ *and* $\psi:[c_{1}, c_{2}] \rightarrow \mathbb{R}$ *be an* *α*-*differentiable function on* $(c_{1}, c_{2})$. *Then the inequality*

2.9 $$\begin{aligned} & \biggl\vert \frac{\psi(c_{1})+\psi(c_{2})}{2}-\frac{\alpha }{c_{2}^{\alpha}-c_{1}^{\alpha}} \int_{c_{1}}^{c_{2}}\psi(\xi)\,d_{\alpha }\xi \biggr\vert \\ &\quad \leq\frac{c_{2}-c_{1}}{2(c_{2}^{\alpha}-c_{1}^{\alpha})} \biggl[ \mathrm{A}_{1}(\alpha) \psi^{\prime} \biggl(\frac{\mathrm{C}_{1}(\alpha )}{\mathrm{A}_{1}(\alpha)} \biggr) +\mathrm{B}_{1}( \alpha)\psi^{\prime} \biggl(\frac{\mathrm{C}_{2}(\alpha )}{\mathrm{B}_{1}(\alpha)} \biggr) \biggr] \end{aligned}$$

*holds if* $\mathrm{D}_{\alpha}(\psi)\in \mathrm{L}_{\alpha}^{1}([c_{1}, c_{2}])$ *and* $\vert \psi^{\prime } \vert ^{q}$ *is concave on* $[c_{1}, c_{2}]$, *where* $\mathrm{A}_{1}(\alpha)$ *and* $\mathrm{B}_{1}(\alpha)$ *are defined as in Theorem* 2.5, *and* $\mathrm{C}_{1}(\alpha)$ *and* $\mathrm{C}_{2}(\alpha)$ *are defined by*

$$ \mathrm{C}_{1}(\alpha)= \biggl[\frac{c_{1}^{\alpha+2}-c_{2}^{\alpha +2}}{(\alpha+2)(c_{1}-c_{2})}-\frac{c_{1}^{\alpha}(c_{1}-c_{2})}{2} \biggr],\quad \quad \mathrm{C}_{2}(\alpha)= \biggl[\frac{c_{2}^{\alpha}(c_{1}+c_{2})}{2}- \frac {c_{1}^{\alpha+2}-c_{2}^{\alpha+2}}{(\alpha+2)(c_{1}-c_{2})} \biggr]. $$

### Proof

It follows from the concavity of $\vert \psi' \vert ^{q}$ and the Hölder mean inequality that

$$\begin{aligned}& \bigl(\theta \bigl\vert \psi^{\prime}(c_{1}) \bigr\vert +(1- \theta) \bigl\vert \psi^{\prime}(c_{2}) \bigr\vert \bigr)^{q}\leq\theta \bigl\vert \psi ^{\prime}(c_{1}) \bigr\vert ^{q}+(1-\theta) \bigl\vert \psi^{\prime }(c_{2}) \bigr\vert ^{q} \leq \bigl\vert \psi^{\prime}\bigl(\theta c_{1}+(1-\theta)c_{2}\bigr) \bigr\vert ^{q}, \\& \bigl\vert \psi^{\prime}\bigl(\theta c_{1}+(1- \theta)c_{2}\bigr) \bigr\vert \geq \theta \bigl\vert \psi^{\prime}(c_{1}) \bigr\vert +(1-\theta) \bigl\vert \psi^{\prime}(c_{2}) \bigr\vert , \end{aligned}$$

which implies that $\vert \psi' \vert $ is also concave. Making use of Lemma 2.1 and the Jensen integral inequality, we have

2.10 $$\begin{aligned} & \biggl\vert \frac{\psi(c_{1})+\psi(c_{2})}{2}-\frac{\alpha }{c_{2}^{\alpha}-c_{1}^{\alpha}} \int_{c_{1}}^{c_{2}}\psi(\xi)\,d_{\alpha }\xi \biggr\vert \\ &\quad = \biggl\vert \frac{c_{2}-c_{1}}{2(c_{2}^{\alpha}-c_{1}^{\alpha})} \biggl[ \int_{0}^{1} \bigl(\bigl(\theta c_{1}+(1- \theta)c_{2}\bigr)^{2\alpha-1}-c_{1}^{\alpha}\bigl( \theta c_{1}+(1-\theta)c_{2}\bigr)^{\alpha-1} \bigr) \\ &\quad\quad{}\times \mathrm{D} _{\alpha}(\psi) \bigl(\theta c_{1}+(1- \theta)c_{2}\bigr)\,d\theta \\ &\quad\quad{} + \int_{0}^{1} \bigl(\bigl(\theta c_{1}+(1- \theta)c_{2}\bigr)^{2\alpha-1}-c_{2}^{\alpha}\bigl( \theta c_{1}+(1-\theta)c_{2}\bigr)^{\alpha-1} \bigr) \mathrm{D}_{\alpha}(\psi) \bigl(\theta c_{1}+(1- \theta)c_{2}\bigr)\,d\theta \biggr] \biggr\vert \\ &\quad \leq\frac{c_{2}-c_{1}}{2(c_{2}^{\alpha}-c_{1}^{\alpha})} \biggl[ \int _{0}^{1} \bigl(\bigl(\theta c_{1}+(1- \theta)c_{2}\bigr)^{\alpha}-c_{1}^{\alpha} \bigr) \bigl\vert \psi ^{\prime}\bigl(\theta c_{1}+(1- \theta)c_{2}\bigr) \bigr\vert \,d\theta \\ &\quad\quad{} + \int_{0}^{1} \bigl(c_{2}^{\alpha}- \bigl(\theta c_{1}+(1-\theta)c_{2}\bigr)^{\alpha} \bigr) \bigl\vert \psi^{\prime}\bigl(\theta c_{1}+(1- \theta)c_{2}\bigr) \bigr\vert \,d\theta \biggr], \end{aligned}$$

2.11 $$\begin{aligned} & \int_{0}^{1} \bigl(\bigl(\theta c_{1}+(1- \theta)c_{2}\bigr)^{\alpha}-c_{1}^{\alpha} \bigr) \bigl\vert \psi ^{\prime}\bigl(\theta c_{1}+(1- \theta)c_{2}\bigr) \bigr\vert \,d\theta \\ &\quad \leq \biggl( \int_{0}^{1} \bigl(\bigl(\theta c_{1}+(1- \theta)c_{2}\bigr)^{\alpha}-c_{1}^{\alpha} \bigr)\,d\theta \biggr) \\ &\quad\quad{} \times\psi^{\prime} \biggl(\frac{\int_{0}^{1} ((\theta c_{1}+(1-\theta)c_{2})^{\alpha}-c_{1}^{\alpha} ) (\theta c_{1}+(1-\theta)c_{2})\,d\theta}{\int_{0}^{1} ((\theta c_{1}+(1-\theta)c_{2})^{\alpha}-c_{1}^{\alpha} )\,d\theta} \biggr) \\ &\quad =\mathrm{A}_{1}(\alpha)\psi^{\prime} \biggl(\frac{\mathrm{C}_{1}(\alpha )}{\mathrm{A}_{1}(\alpha)} \biggr), \end{aligned}$$

2.12 $$\begin{aligned} & \int_{0}^{1} \bigl(c_{2}^{\alpha}- \bigl(\theta c_{1}+(1-\theta)c_{2}\bigr)^{\alpha} \bigr) \bigl\vert \psi^{\prime}\bigl(\theta c_{1}+(1- \theta)c_{2}\bigr) \bigr\vert \,d\theta \\ &\quad \leq \biggl(c_{2}^{\alpha}- \int_{0}^{1} \bigl(\bigl(\theta c_{1}+(1- \theta)c_{2}\bigr)^{\alpha} \bigr)\,d\theta \biggr) \\ &\quad\quad{} \times\psi^{\prime} \biggl(\frac{\int_{0}^{1} (c_{2}^{\alpha}-(\theta c_{1}+(1-\theta)c_{2})^{\alpha} )(\theta c_{1}+(1-\theta)c_{2})\,d\theta}{ \int_{0}^{1} (c_{2}^{\alpha}-(\theta c_{1}+(1-\theta)c_{2})^{\alpha} )\,d\theta} \biggr) \\ &\quad =\mathrm{B}_{1}(\alpha)\psi^{\prime} \biggl(\frac{\mathrm{C}_{2}(\alpha )}{\mathrm{B}_{1}(\alpha)} \biggr). \end{aligned}$$

Therefore, inequality (2.9) follows easily from (2.10)-(2.12). □

### Remark 2.8

Let $\alpha=1$. Then inequality (2.9) leads to

$$\begin{aligned} & \biggl\vert \frac{\psi(c_{1})+\psi(c_{2})}{2}-\frac{1}{c_{2}-c_{1}} \int _{c_{1}}^{c_{2}}\psi(\xi)\,d\xi \biggr\vert \\ &\quad \leq\frac{c_{2}-c_{1}}{4} \biggl[\psi^{\prime} \biggl(\frac {2c_{2}^{2}-c_{1}^{2}+5c_{1}c_{2}}{3(c_{2}-c_{1})} \biggr) +\psi^{\prime} \biggl(\frac{c_{2}^{2}-2c_{1}^{2}+c_{1} c_{2}}{3(c_{2}-c_{1})} \biggr) \biggr]. \end{aligned}$$

## Applications to special means of real numbers

Let $\alpha\in(0,1]$, $r\in\mathbb{R}$, $r\neq0, -\alpha$ and $a, b>0$ with $a\neq b$. Then the arithmetic mean $\mathrm{A}(a, b)$, logarithmic mean $\mathrm{L}(a, b)$ and $(\alpha, r)$th generalized logarithmic mean $\mathrm{L}_{(\alpha, r)}(a,b)$ of *a* and *b* are defined by

$$ \mathrm{A}(a,b)=\frac{a+b}{2}, \quad \quad\mathrm{L}(a,b)=\frac{a-b}{\log a-\log b}, \quad\quad \mathrm{L}_{(\alpha, r)}(a,b)= \biggl[\frac{\alpha (b^{\alpha+r}-a^{\alpha+r} )}{(\alpha+r) (b^{\alpha}-a^{\alpha} )} \biggr]^{1/r}, $$

respectively. Then from Theorems 2.3 and 2.5 together with the convexities of the functions $\xi\rightarrow\xi^{r}$ and $\xi\rightarrow1/\xi$ on the interval $(0, \infty)$ we get several new inequalities for the arithmetic, logarithmic and generalized logarithmic means as follows.

### Theorem 3.1

*Let* $c_{1},c_{2}\in\mathbb{R}$ *with* $0< c_{1}< c_{2}$, $r>1$, $q>1$ *and* $\alpha\in(0, 1]$. *Then we have*

$$\begin{aligned}& \bigl\vert \mathrm{A}\bigl(c_{1}^{r},c_{2}^{r} \bigr)-\mathrm{L}^{r}_{(\alpha ,r)}(c_{1},c_{2}) \bigr\vert \\& \quad \leq\frac{r(c_{2}-c_{1}) (5c_{2}^{\alpha}-7c_{1}^{\alpha }+c_{1}c_{2}^{\alpha-1}+c_{1}^{\alpha-1}c_{2} )}{ 12(c_{2}^{\alpha}-c_{1}^{\alpha})}\mathrm{A}\bigl( \vert c_{1} \vert ^{r-1}, \vert c_{2} \vert ^{r-1}\bigr), \\& \bigl\vert \mathrm{A}\bigl(c_{1}^{r},c_{2}^{r} \bigr)-\mathrm{L}^{r}_{(\alpha ,r)}(c_{1},c_{2})) \bigr\vert \\& \quad \leq \frac{r(c_{2}-c_{1})}{2(c_{2}^{\alpha}-c_{1}^{\alpha})} \bigl[ \bigl(\mathrm{A}_{1} (\alpha) \bigr)^{1-1/q} \bigl\{ \mathrm{A}_{2}(\alpha) \vert c_{1} \vert ^{(r-1)q} +\mathrm{A}_{3}(\alpha) \vert c_{2} \vert ^{(r-1)q} \bigr\} ^{1/q} \\& \quad\quad{} + \bigl(\mathrm{B}_{1}(\alpha) \bigr)^{1-1/q} \bigl\{ \mathrm{B}_{2} (\alpha) \vert c_{1} \vert ^{(r-1)q}+\mathrm{B}_{3}(\alpha ) \vert c_{2} \vert ^{(r-1)q} \bigr\} ^{1/q} \bigr], \\& \bigl\vert \mathrm{A}\bigl(c_{1}^{-1},c_{2}^{-1} \bigr)-\mathrm{L}^{-1}_{(\alpha ,-1)}(c_{1},c_{2}) \bigr\vert \leq \frac{(c_{2}-c_{1}) (5c_{2}^{\alpha}-7c_{1}^{\alpha }+c_{1}c_{2}^{\alpha-1}+c_{1}^{\alpha-1}c_{2} )}{ 12(c_{2}^{\alpha}-c_{1}^{\alpha})}\mathrm{A}\bigl(c_{1}^{-2}, c_{2}^{-2}\bigr), \\& \bigl\vert \mathrm{A}\bigl(c_{1}^{-1},c_{2}^{-1} \bigr)-\mathrm{L}^{-1}_{(\alpha, -1)}(c_{1},c_{2}) \bigr\vert \leq \frac{c_{2}-c_{1}}{2(c_{2}^{\alpha}-c_{1}^{\alpha})} \bigl[ \bigl(\mathrm {A}_{1}( \alpha) \bigr)^{1-1/q} \bigl\{ \mathrm{A}_{2}(\alpha) \vert c_{1} \vert ^{-2q}+\mathrm {A}_{3}(\alpha) \vert c_{2} \vert ^{-2q} \bigr\} ^{1/q} \\& \hphantom{\bigl\vert \mathrm{A}\bigl(c_{1}^{-1},c_{2}^{-1} \bigr)-\mathrm{L}^{-1}_{(\alpha, -1)}(c_{1},c_{2}) \bigr\vert \leq}{}+ \bigl(\mathrm{B}_{1}(\alpha) \bigr)^{1-1/q} \bigl\{ \mathrm {B}_{2}(\alpha) \vert c_{1} \vert ^{-2q} + \mathrm{B}_{3}(\alpha) \vert c_{2} \vert ^{-2q} \bigr\} ^{\frac {1}{q}} \bigr], \end{aligned}$$

*where* $\mathrm{A}_{1}(\alpha)$, $\mathrm{A}_{2}(\alpha)$, $\mathrm{A}_{3}(\alpha)$, $\mathrm{B}_{1}(\alpha)$, $\mathrm{B}_{2}(\alpha)$ *and* $\mathrm{B}_{3}(\alpha)$ *are defined as in Theorem* 2.5.

## Applications to the trapezoidal formula

Let Δ be a division $c_{1}=\xi_{0}<\xi_{1}<\cdots<\xi_{n-1}<\xi_{n}=c_{2}$ of the interval $[c_{1}, c_{2}]$ and consider the quadrature formula

$$ \int_{c_{1}}^{c_{2}}\psi(\xi)\,d_{\alpha}\xi= \mathrm{T}_{\alpha}(\psi, \Delta)+\mathrm{E}_{\alpha}(\psi, \Delta), $$

where

$$ \mathrm{T}_{\alpha}(\psi, \Delta)=\sum_{i=0}^{n-1} \frac{\psi(\xi_{i})+\psi(\xi_{i+1})}{ 2}\frac{ (\xi^{\alpha}_{i+1}-\xi^{\alpha}_{i} )}{\alpha} $$

is the trapezoidal version and $\mathrm{E}_{\alpha}(\psi, \Delta)$ denotes the associated approximation error. In this section, we are going to derive several new error estimations for the trapezoidal formula.

### Theorem 4.1

*Let* $\alpha\in(0, 1]$, $c_{1}, c_{2}\in\mathbb{R}$ *with* $0\leq c_{1} < c_{2}$, $\psi:[c_{1}, c_{2}]\rightarrow\mathbb{R}$ *be an* *α*-*differentiable function on* $(c_{1}, c_{2})$ *and* Δ *be a division* $c_{1}=\xi_{0}<\xi_{1}<\cdots<\xi_{n-1}<\xi_{n}=c_{2}$ *of the interval* $[c_{1}, c_{2}]$. *Then the inequality*

$$ \bigl\vert \mathrm{E}_{\alpha}(\psi, \Delta) \bigr\vert \leq \frac{1}{12\alpha}\max\bigl\{ \bigl\vert \psi^{\prime}(c_{1}) \bigr\vert , \bigl\vert \psi^{\prime}(c_{2}) \bigr\vert \bigr\} \sum_{i=0}^{n-1}(\xi _{i+1}- \xi_{i}) \bigl(5\xi_{i+1}^{\alpha}-7 \xi_{i}^{\alpha}+\xi_{i}\xi_{i+1}^{\alpha -1}+ \xi_{i}^{\alpha-1}\xi_{i+1} \bigr) $$

*holds if* $\mathrm{D}_{\alpha}(\psi)\in \mathrm{L}_{\alpha}^{1}([c_{1},c_{2}])$ *and* $\vert \psi^{\prime } \vert $ *is convex on* $[c_{1},c_{2}]$.

### Proof

Applying Theorem 2.3 on the subinterval $[\xi_{i},\xi _{i+1}]$ $(i= 0, 1,\ldots, n - 1)$ of the division Δ, we have

4.1 $$\begin{aligned} & \biggl\vert \frac{\psi(\xi_{i})+\psi(\xi_{i+1})}{2}\frac{(\xi^{\alpha }_{i+1}-\xi^{\alpha}_{i})}{ \alpha}- \int_{\xi_{i}}^{\xi_{i+1}}\psi(\xi)\,d_{\alpha}\xi \biggr\vert \\ &\quad \leq\frac{(\xi_{i+1}-\xi_{i})}{2\alpha} \biggl[\frac{( \vert \psi^{\prime}(\xi_{i}) \vert + \vert \psi^{\prime}(\xi_{i+1}) \vert ) (5\xi_{i+1}^{\alpha}-7\xi _{i}^{\alpha}+ \xi_{i}\xi_{i+1}^{\alpha-1}+\xi_{i}^{\alpha-1}\xi_{i+1} )}{12} \biggr]. \end{aligned}$$

It follows from (4.1) and the convexity of $\vert \psi^{\prime}(\xi ) \vert $ on the interval $[c_{1}, c_{2}]$ that

$$\begin{aligned}& \bigl\vert \mathrm{E}_{\alpha}(\psi, \Delta) \bigr\vert = \biggl\vert \mathrm{T}_{\alpha}(\psi, \Delta)- \int_{c_{1}}^{c_{2}}\psi(\xi)\,d_{\alpha}\xi \biggr\vert \\& \quad = \Biggl\vert \sum_{i=0}^{n-1} \biggl[ \frac{\psi(\xi_{i})+\psi(\xi_{i+1})}{2} \frac{ (\xi^{\alpha}_{i+1}-\xi^{\alpha}_{i} )}{\alpha}- \int _{\xi_{i}}^{\xi_{i+1}}\psi(\xi)\,d_{\alpha}\xi \biggr] \Biggr\vert \\& \quad \leq \sum_{i=0}^{n-1} \biggl\vert \frac{\psi(\xi_{i})+\psi(\xi_{i+1})}{2} \frac{ (\xi^{\alpha}_{i+1}-\xi^{\alpha}_{i} )}{\alpha}- \int _{\xi_{i}}^{\xi_{i+1}}\psi(\xi)\,d_{\alpha}\xi \biggr\vert \\& \quad \leq \frac{1}{2\alpha}\sum_{i=0}^{n-1}( \xi_{i+1}-\xi_{i}) \biggl[\frac{( \vert \psi^{\prime}(\xi_{i}) \vert + \vert \psi^{\prime}(\xi_{i+1}) \vert ) (5\xi_{i+1}^{\alpha}-7\xi _{i}^{\alpha} +\xi_{i}\xi_{i+1}^{\alpha-1}+\xi_{i}^{\alpha-1}\xi_{i+1} )}{12} \biggr] \\& \quad = \frac{1}{12\alpha}\sum_{i=0}^{n-1}( \xi_{i+1}-\xi_{i}) \biggl[\frac{( \vert \psi^{\prime}(\xi_{i}) \vert + \vert \psi^{\prime}(\xi_{i+1}) \vert ) (5\xi_{i+1}^{\alpha}-7\xi _{i}^{\alpha} +\xi_{i}\xi_{i+1}^{\alpha-1}+\xi_{i}^{\alpha-1}\xi_{i+1} )}{2} \biggr] \\& \quad \leq \frac{1}{12\alpha}\sum_{i=0}^{n-1}( \xi_{i+1}-\xi_{i}) \bigl(5\xi _{i+1}^{\alpha}-7 \xi_{i}^{\alpha} +\xi_{i}\xi_{i+1}^{\alpha-1}+ \xi_{i}^{\alpha-1}\xi_{i+1} \bigr)\max\bigl\{ \bigl\vert \psi^{\prime}(\xi_{i}) \bigr\vert , \bigl\vert \psi^{\prime}(\xi_{i+1}) \bigr\vert \bigr\} \\& \quad \leq \frac{1}{12\alpha}\max\bigl\{ \bigl\vert \psi^{\prime}(c_{1}) \bigr\vert , \bigl\vert \psi^{\prime}(c_{2}) \bigr\vert \bigr\} \sum_{i=0}^{n-1}(\xi _{i+1}- \xi_{i}) \bigl(5\xi_{i+1}^{\alpha}-7 \xi_{i}^{\alpha}+\xi_{i}\xi_{i+1}^{\alpha -1}+ \xi_{i}^{\alpha-1}\xi_{i+1} \bigr). \end{aligned}$$

 □

Making use of arguments analogous to the proof of Theorem 4.1, we get Theorem 4.2 immediately.

### Theorem 4.2

*Let* $\alpha\in(0, 1]$, $q>1$, $c_{1}, c_{2}\in\mathbb{R}$ *with* $0\leq c_{1} < c_{2}$, $\psi:[c_{1}, c_{2}]\rightarrow\mathbb{R}$ *be an* *α*-*differentiable function on* $(c_{1}, c_{2})$ *and* Δ *be a division* $c_{1}=\xi_{0}<\xi_{1}<\cdots<\xi_{n-1}<\xi_{n}=c_{2}$ *of the interval* $[c_{1}, c_{2}]$. *Then the inequality*

$$\begin{aligned} \bigl\vert \mathrm{E}_{\alpha}(\psi, \Delta) \bigr\vert &\leq\sum _{i=0}^{n-1}\frac{(\xi_{i+1}-\xi_{i})}{2\alpha} \bigl[ \bigl( \mathrm{A}_{1}(\alpha) \bigr)^{1-1/q} \bigl\{ \mathrm{A}_{2}(\alpha) \bigl\vert \psi^{\prime}( \xi_{i}) \bigr\vert ^{q}+\mathrm{A}_{3}(\alpha) \bigl\vert \psi^{\prime}(\xi _{i+1}) \bigr\vert ^{q} \bigr\} ^{1/q} \\ &\quad{} + \bigl(\mathrm{B}_{1}(\alpha) \bigr)^{1-1/q} \bigl\{ \mathrm {B}_{2}(\alpha) \bigl\vert \psi^{\prime}(\xi_{i}) \bigr\vert ^{q} +\mathrm{B}_{3}(\alpha) \bigl\vert \psi^{\prime}(\xi_{i+1}) \bigr\vert ^{q} \bigr\} ^{1/q} \bigr] \end{aligned}$$

*holds if* $\mathrm{D}_{\alpha}(\psi)\in \mathrm{L}_{\alpha}^{1}([c_{1},c_{2}])$ *and* $\vert \psi^{\prime } \vert ^{q}$ *is convex on* $[c_{1},c_{2}]$, *where* $\mathrm{A}_{1}(\alpha)$, $\mathrm{A}_{2}(\alpha)$, $\mathrm{A}_{3}(\alpha)$, $\mathrm{B}_{1}(\alpha)$, $\mathrm{B}_{2}(\alpha)$ *and* $\mathrm{B}_{3}(\alpha)$ *are defined as in Theorem* 2.5.

## Conclusion

In this work, we find an identity and several Hermite-Hadamard type inequalities for conformable fractional integrals, present some new inequalities for the arithmetic, logarithmic and generalized logarithmic means of two positive real numbers and provide the error estimations for the trapezoidal formula.
